# Inhibition of VDAC1 Rescues A*β*_1-42_-Induced Mitochondrial Dysfunction and Ferroptosis via Activation of AMPK and Wnt/*β*-Catenin Pathways

**DOI:** 10.1155/2023/6739691

**Published:** 2023-02-10

**Authors:** Xinpei Zhou, Ximin Tang, Tao Li, Dandan Li, Zhiting Gong, Xiujun Zhang, Yanjiao Li, Jianhua Zhu, Yong Wang, Bensi Zhang

**Affiliations:** ^1^Department of Human Anatomy, College of Basic Medicine, Dali University, Xiaguan Campus of Dali University, Wanhua Road, Dali City, Yunnan Province, China; ^2^Department of Computer Network, College of Mathematics and Computer Science, Dali University, Ancient City Campus of Dali University, Wanhua Road, Dali City, Yunnan Province, China; ^3^Department of Internal Medicine-Neurology, College of Clinical Medicine, Dali University, Xiaguan Campus of Dali University, Wanhua Road, Dali City, Yunnan Province, China

## Abstract

Beta-amyloid (A*β*) accumulation in the brains of Alzheimer's disease (AD) patients leads to mitochondrial dysfunction and ferroptosis in neurons. Voltage-dependent anion channel 1 (VDAC1) is a major protein in the mitochondrial outer membrane. It has been reported that VDAC1 associated with mitochondrial dysfunction and ferroptosis. However, the mechanism by which VDAC1 regulates mitochondrial dysfunction and ferroptosis of neurons in AD remains unclear. This study is aimed at investigating the mechanism of action of VDAC1 in mitochondrial dysfunction and ferroptosis in neurons of the AD model. In this study, we determined cell viability after treatment with A*β*_1-42_ via the MTT assay. The SOD, MDA, ROS, and MMP production was measured via the SOD kit, MDA kit, DCFDA staining, and JC-1 staining. The memory abilities of mice were detected via the Morris water maze test. The expression of AMPK/mTOR, Wnt/*β*-catenin, and GPX4 regulated by VDAC1 was detected via western blotting. Our present study showed that PC12 cells had decreased cell viability, increased LDH release, and decreased GPX4 expression after A*β*_1-42_ treatment. Meanwhile, A*β*_1-42_ induced MMP and SOD downregulation and increased MDA and ROS generation in PC12 cells. In addition, the expression of VDAC1 is increased in the brain tissue of AD mice and A*β*_1-42_-treated PC12 cells. Further investigation of the role of VDAC1 in regulating AD found that all effects induced by A*β*_1-42_ were reversed by inhibition of VDAC1. Additionally, inhibition of VDAC1 activates the AMPK/mTOR and Wnt/*β*-catenin pathways. Taken together, these findings demonstrate that inhibition of VDAC1 alleviates mitochondrial dysfunction and ferroptosis in AD neurons by activating AMPK/mTOR and Wnt/*β*-catenin.

## 1. Introduction

AD is a common age-related neurodegenerative disease characterized by impairment of memory and cognitive function [1–5]. The deposition of amyloid *β* (A*β*) protein and tau protein is currently considered a neuropathological hallmark of AD [6]. Among them, the deposition of A*β* can lead to neuronal dysfunction and death in the brain [7]. Despite several strategies and interventions having been adopted for the pathological characteristics of animal models and patients, alleviating neuronal damage remains a huge challenge for current AD treatment.

Recent studies have found that neuronal death in AD appears to be associated with a decline in mitochondrial function [8]. Multiple mitochondrial alterations in AD neurons impair mitochondrial metabolism and reduce mitochondrial function leading to neuronal death [9]. Mitochondria play a critical role in neuronal cell survival as efficient integrators and coordinators of energy metabolism and cell death [10]. Impaired mitochondrial biosynthesis promotes mitochondrial dysfunction in cells [11]. Previous studies have shown that mitochondrial dysfunction is associated with the development of nearly all neurological disorders, including AD [10, 12]. In AD patients, altered mitochondrial structure and mitochondrial enzymes have been observed [13]. In addition, the accumulation of A*β* impairs the physiological function of mitochondria, disrupts the mitochondrial respiratory chain complex, increases the accumulation of ROS, and reduces ATP production and mitochondrial membrane potential (MMP), initiating mitochondrial dysfunction [14]. However, the mechanism leading to mitochondrial dysfunction in AD is still unclear.

Iron plays a key role for neuronal proliferation, myelination, neurotransmitter synthesis, and energy metabolism during brain development [15, 16]. Steady uptake, distribution, and efflux of iron in neurons regulate neuronal survival and affect neuronal function [17]. When cellular iron homeostasis is disrupted, dysregulated accumulation of iron leads to cell death, termed as ferroptosis [18]. Ferroptosis has been reported to be associated with neurodegenerative disease (i.e., AD, Huntington's disease, and Parkinson's disease) and has been shown to be a specific target for the treatment of AD [19]. In addition, during the process of ferroptosis, the accumulation of lipid peroxidation also promotes the damage of mitochondrial function and exacerbates mitochondrial dysfunction [20]. Current studies have confirmed that ferroptosis and mitochondrial dysfunction may be associated with neuronal death in AD. However, direct evidence is still lacking.

VDAC1, as a key regulator of mitochondrial function, is ubiquitous in the outer mitochondrial membrane [21]. Researchers have demonstrated VDAC1 overexpression in the brains of AD patients and AD transgenic mice [22]. Simultaneously, VDAC1 levels are elevated in the brains of AD patients, reflecting oxidative damage to AD neurons by VDAC1 [23] and affecting neuronal mitochondrial function and metabolite homeostasis [24]. In addition, studies have found that inhibition of VDAC1 can alleviate ferroptosis by protecting mitochondria [25]. However, the role of VDAC1 in AD development by regulating neuronal mitochondrial function and ferroptosis remains unclear.

In this study, we found that the expression of VDAC1 is increased in the brains of an AD mouse model as well as in A*β*_1-42_-induced PC12 cells (and SH-SY5Y cells). By using ferroptosis inhibitors and detecting changes in ferroptosis-related protein expressions and mitochondrial function, we found that VDAC1 is closely related to A*β*_1-42_-induced mitochondrial dysfunction and ferroptosis in PC12 cells. Meanwhile, AMPK/mTOR and Wnt/*β*-catenin were inhibited in response to A*β*_1-42_. Therefore, the purpose of this study was to investigate whether VDAC1 upregulated by A*β*_1-42_ action inhibits AMPK/mTOR and Wnt/*β*-catenin downregulates GPX4 to promote mitochondrial dysfunction and ferroptosis in PC12 cells.

## 2. Materials and Methods

### 2.1. Reagents and Antibodies

A*β*_1-42_ was bought from the Peptide Institute (Osaka, Japan). VBIT-4 was bought from ChemPartner (Chengdu, China). Ferrostatin-1, Emricasan, and Necrostatin-1 were purchased from Selleckchem (Selleck Chemicals; USA). Antibodies were purchased from Santa Cruz Biotechnology (Santa Cruz, CA, USA). PC12 cells and SH-SY5Y cells were obtained from the Bena Culture Collection (Beijing, China).

### 2.2. Animal Model

Eight-month-old APP/PS1 mice were bought from HFK (Bioscience Co., Ltd., Beijing), and C57BL/6J wild-type (WT) mice served as the controls [26]. All animal procedures were approved by the Laboratory Animal Committee of Dali University (no. 2021-PZ-056). In the VDAC1 inhibitor VBIT-4 treatment group, VBIT-4 was injected intraperitoneally (25 mg/kg) for 4 weeks.

### 2.3. Cell Culture and Treatments

PC12 cells and SH-SY5Y cells were cultured in Dulbecco's modified Eagle's medium- (DMEM-) supplemented fetal bovine serum (10% FBS) (Gibco, Carlsbad, CA) and 1% penicillin/streptomycin (Thermo Fisher Scientific, Inc., USA). All cells were cultured at 37°C under humidified air containing 5% CO_2_. The cells were stimulated with A*β*_1-42_ (5 *μ*M). The cells were pretreated with Ferrostatin-1 (5 *μ*M; 30 min) before the stimulation of A*β*_1-42_.

### 2.4. Cell Viability Assay

The MTT assay was employed to determine the viability of PC12 and SH-SY5Y cells treated with A*β*_1-42_, Ferrostatin-1, and si-VDAC1/si-NT. Briefly, cells were seeded in 96-well plates and were treated with A*β*_1-42_, Ferrostatin-1, and si-VDAC1/si-NT. Next, 50 *μ*L of MTT was added into every well for another 4 h at 37°C. The absorbance was measured at 490 nm using an automatic microplate reader (Thermo Scientific, Waltham, MA, USA).

### 2.5. Western Blotting Assay

For mouse brain tissue, mice were killed by decapitation, the brain tissues of the prefrontal cortex were obtained from whole brains, and the tissues were homogenated with a sample buffer supplemented with a phosphatase inhibitor and protease inhibitor cocktail on ice and boiled. For cells, cell proteins were extracted in 1x Laemmli lysis buffer and boiled. Protein concentration was determined by using a Bio-Rad protein assay reagent (Hercules, CA, USA). An equal quantity of proteins was separated by 10% SDS-PAGE gel and transferred to a polyvinylidene difluoride membrane (Merck Millipore, Burlington, MA, USA). The membranes were incubated with a primary antibody overnight at 4°C. Then, the membranes were incubated with horseradish peroxidase-conjugated secondary antibodies for 1 h. The immunoblots were visualized using an ECL chemiluminescence system (Millipore Corporation, Billerica, MA, USA), and the blots were analyzed using ImageJ software (NIH, Bethesda, MD, USA).

### 2.6. Lactate Dehydrogenase Assay

Cell death was evaluated and detected using an LDH assay kit (Beyotime, China). Briefly, cells were collected after A*β*_1-42_, Fer-1, or si-VDAC1/si-NT treatment, and the supernatant was separated, following the manufacturer's instructions. Then, cell supernatants were incubated with an LDH reaction mix for 30 min, and the absorbance was all measured at 450 nm.

### 2.7. Determination of Mitochondrial Membrane Potential

Intracellular MMP production was measured by staining with tetraethylbenzimidazolylcarbocyanine iodide (JC-1, Beyotime, China). Briefly, cells were treated via A*β*_1-42_, Fer-1, or si-VDAC1/si-NT and incubated with 500 *μ*L JC-1 (10 mg/mL) for 20 min (protected from light). Next, the culture medium was aspirated, and the cells were washed with PBS. Images of JC-1 staining were observed using fluorescence microscopy [27].

### 2.8. Oxidative Stress Measurement

#### 2.8.1. Detection of MDA and SOD

Cells were treated via A*β*_1-42_, Fer-1, or si-VDAC1/si-NT. Then, superoxide dismutase (SOD) activity and malondialdehyde (MDA) concentration in cells were determined according to the SOD kit and MDA kit (Beyotime, Shanghai, China). The OD values were determined by a full-wavelength microplate photometer (Thermo Fisher, MA, USA).

#### 2.8.2. DCFH-DA Staining

ROS generation was measured via DCFH-DA staining to detect intracellular hydrogen peroxide and oxidative stress (Beyotime, Shanghai, China). In brief, PC12 cells in 6-well plates were treated via A*β*_1-42_, Fer-1 or si-VDAC1/si-NT. Then, 10 *μ*M DCFH-DA was added into cells and reacted for 15 min. After washing twice with DMEM, the cells were examined via confocal microscopy (Nikon, Japan).

### 2.9. Morris Water Maze (MWM) Test

The Morris water maze was used to assess the learning and memory abilities of mice as described by Bao et al. [28]. Briefly, fill a large circular pool with water and place a circular escape platform; mice were trained to find the platform in the pool. Track the movement trajectories of mice in the pool using tracking equipment. The trial ended when the mice managed to locate the submerged platform. The escape latency and swimming path to find the platform were recorded and analyzed.

### 2.10. Statistical Analysis

All data were presented as mean ± SD. *t*-test and one-way analysis of variance (ANOVA) were used to compare cells and animals with different treatments. A value of *p* < 0.05 or *p* < 0.01 was considered as statistically significant.

## 3. Results

### 3.1. A*β*_1-42_ Effects on Cell Viability and Cytotoxicity and VDAC1, GPX4, and FTH1 Expressions in PC12 and SH-SY5Y Cells

**Figure 1:**
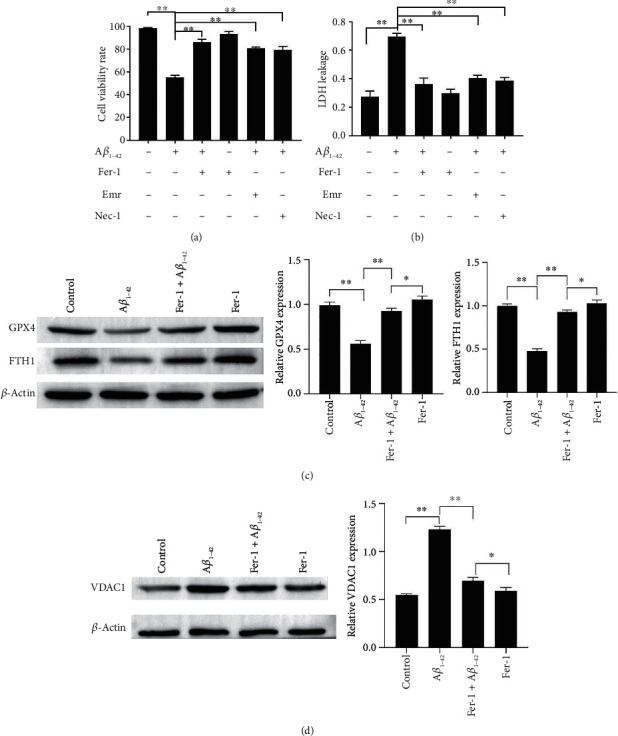
A*β*_1-42_ effects on cell viability and cytotoxicity and VDAC1, GPX4, and FTH1 expressions in PC12 cells. PC12 cell viability was measured via the MTT assay (a). The LDH release was measured via the LDH assay kit (b). The expressions of GPX4, FTH1, and VDAC1 in PC12 cells were measured by western blotting (c, d). Asterisks indicate statistical significance (^∗^*p* < 0.05, ^∗∗^*p* < 0.01).

PC12 cells and SH-SY5Y cells are commonly used as cellular models of AD after A*β*_1-42_ treatment [29, 30]. We validated cell viability and cytotoxicity using the MTT assay and LDH release, respectively, after A*β*_1-42_ treatment. MTT and LDH release assays revealed that 24 h of exposure of PC12 and SH-SY5Y cells to A*β*_1-42_ (5 *μ*M) decreased cell viability significantly and showed obvious toxic effects, while treatment with Fer-1 (5 *μ*M; 30 min) partially reduced cell death and LDH release (Figures 1(a) and 1(b) and Figures S1A-S1B). In addition, necrosis and apoptosis have also been associated with A*β*-induced neuronal cell viability and toxicity [28]. We examined the effects of necrosis and apoptosis on A*β*-induced cell viability and toxicity using an inhibitor of apoptosis (Emricasan; 5 *μ*M; 30 min) and necrosis (Nec-1; 10 *μ*M; 30 min) as controls. MTT and LDH release assays revealed that all three of these inhibitors partially reduced cell death and LDH release, while Fer-1 showed the best preventive effects (Figures 1(a) and 1(b) and Figures S1A-S1B). GPX4 and FTH are a central regulator of ferroptosis [31]; western blot analysis indicated that A*β*_1-42_ treatment decreased the expression of GPX4 and FTH1. Furthermore, Fer-1 restored the decreased expression of GPX4 and FTH1 (Figure 1(c) and Figure S1C). These results suggested that A*β*_1-42_ induced ferroptosis in PC12 cells and SH-SY5Y cells. We have confirmed that A*β*_1-42_ induced ferroptosis on two types of nerve cells, and we will focus on PC12 cells in subsequent experiments.

Ferroptosis is characterized by iron-dependent accumulation of lipid peroxides, a process associated with mitochondrial dysfunction [32]. VDAC1 is the main protein on the mitochondrial outer membrane [25]. Western blotting results showed that A*β*_1-42_ upregulated the expression of VDAC1. In contrast, Fer-1 could inhibit the expression of VDAC1 increased by A*β*_1-42_ (Figure 1(d) and Figure S1D). These results suggested that VDAC1 may be involved in ferroptosis.

### 3.2. A*β*_1-42_ Effects on Mitochondrial Dysfunction in Cells

**Figure 2:**
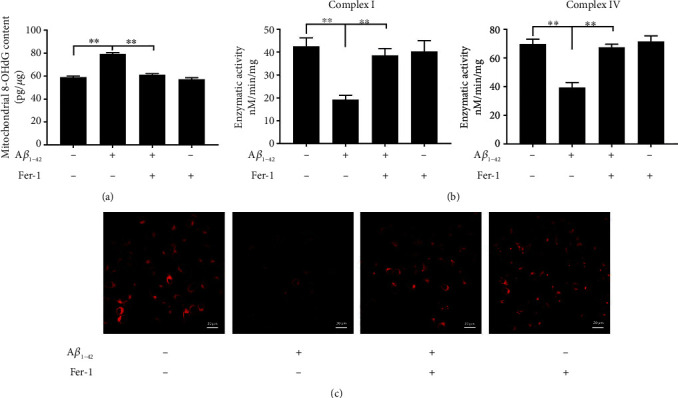
A*β*_1-42_ induces mitochondrial dysfunction. The levels of 8-OHdG were measured via ELISA (a). The levels of mitochondrial respiratory chain complexes I and IV were measured using a complex I enzyme activity assay kit and a cytochrome c oxidase assay kit (b). Mitochondrial membrane potential (ΔΨm) was detected using JC-1 staining, 100x (c). Asterisks indicate statistical significance (^∗^*p* < 0.05, ^∗∗^*p* < 0.01).

We further examined the relationship between ferroptosis and mitochondrial function after PC12 cells were exposed to A*β*_1-42_. Mitochondrial complex activity is related to mitochondrial function [33]; our results in detecting the mitochondrial complex activity indicated that A*β*_1-42_ treatment resulted in remarkable reductions in mitochondrial complex I and IV activities, while Fer-1 treatment restored mitochondrial complex I and IV activity. In addition, A*β*_1-42_ induced elevated levels of 8-OHdG, a marker of mitochondrial oxidative damage. In contrast, Fer-1 treatment restored the levels of 8-OHdG (Figures 2(a) and 2(b)). The mitochondrial membrane potential (MMP) is an important indicator of mitochondrial function [34]. To confirm the effect of Fer-1 on mitochondria protection, we assayed MMP using JC-1. The results showed that MMP was significantly degraded by A*β*_1-42_, and Fer-1 treatment significantly recovered the MMP levels decreased by A*β*_1-42_ (Figure 2(c)). These results indicated the alleviation effects of the Fer-1 on PC12 cell mitochondrial dysfunction induced by A*β*_1-42_.

### 3.3. Inhibition of VDAC1 Restored the Cell Viability, Cytotoxicity, GPX4, and FTH1 Expression Decreased by A*β*_1-42_ and Recovered the MMP Levels Decreased by A*β*_1-42_

**Figure 3:**
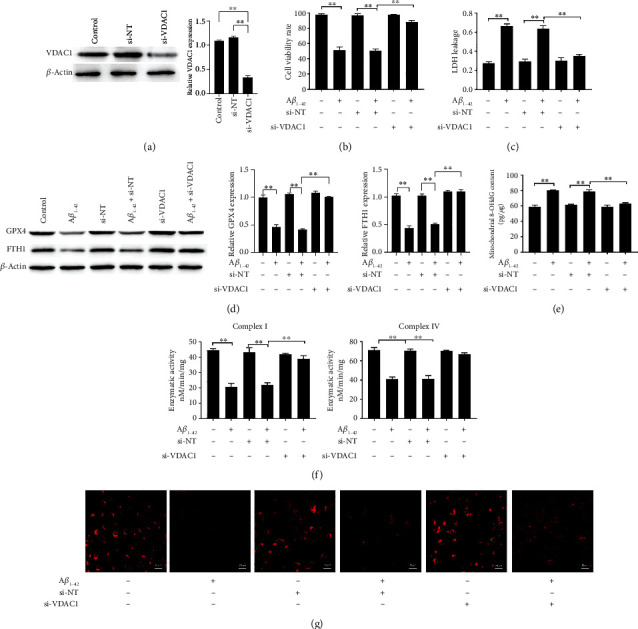
Inhibition of VDAC1 restored the cell viability, cytotoxicity, GPX4, and FTH1 expression decreased by A*β*_1-42_ and recovered the MMP levels decreased by A*β*_1-42_. The expressions of GPX4, FTH1, and VDAC1 in PC12 cells were measured via western blotting (a, d). PC12 cell viability was measured via the MTT assay (b). The LDH release was measured via the LDH assay kit (c). The levels of 8-OHdG were measured via ELISA (e). The levels of mitochondrial respiratory chain complexes I and IV were measured using a complex I enzyme activity assay kit and a cytochrome c oxidase assay kit (f). Mitochondrial membrane potential (ΔΨm) was detected using JC-1 staining, 100x (g). Asterisks indicate statistical significance (^∗^*p* < 0.05, ^∗∗^*p* < 0.01).

Western blot results showed that VDAC1 expression was decreased by si-VDAC (si-hVDAC1-2/A) transfection to PC12 cells (48 h) (Figure 3(a)). In addition, A*β*_1-42_-induced decreased cell viability and increased LDH release were reversed by transfection si-VDAC1 (Figures 3(b) and 3(c)). Simultaneously, the expressions of GPX4 and FTH1 were decreased by A*β*_1-42_, which was restored by transfection si-VDAC1 (Figure 3(d)). Transfection of nontargeting siRNA (si-NT) did not affect VDAC1 expression, cell viability, cytotoxic release, and GPX4 and FTH1 expressions. Transfection with si-VDA restored the activity of complexes I and IV and restored the levels of 8-OHdG (Figures 3(e) and 3(f)). Simultaneously, transfection with si-VDAC significantly restored MMP dysfunction (Figure 3(g)).

### 3.4. Effects of VDAC1 Inhibition on A*β*_1-42_-Induced Cellular Oxidative Stress

**Figure 4:**
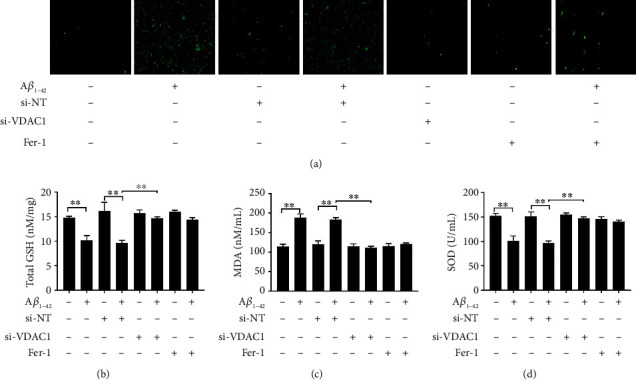
A*β*_1-42_ induces oxidative stress. Quantity analysis of intensity of the DCFDA staining, 100x (a). The levels of GSH were measured via the GSH kit (b). The levels of SOD and MDA were measured via assay kits (c, d). Asterisks indicate statistical significance (^∗^*p* < 0.05, ^∗∗^*p* < 0.01).

Mitochondrial damage causes ROS generation to be involved in ferroptosis [35]. To investigate the ROS-scavenging activity in PC12 cells, DCFH-DA was used. The results showed that si-VDAC1 and Fer-1 treatment could decrease ROS generation induced by A*β*_1-42_ (Figure 4(a)). In addition, A*β*_1-42_ treatment increased MDA expression and decreased GSH and SOD levels. In contrast, si-VDAC1 and Fer-1 effects restored the GSH and SOD levels decreased by A*β*_1-42_ and decreased MDA expression (Figures 4(b)–4(d)). These results suggested that si-VDAC1 prevents oxidative stress induced by A*β*_1-42_, same as the Fer-1 treatment effects.

### 3.5. Inhibition of VDAC1 Effects on Cognitive Function in APP/PS1 Mice

**Figure 5:**
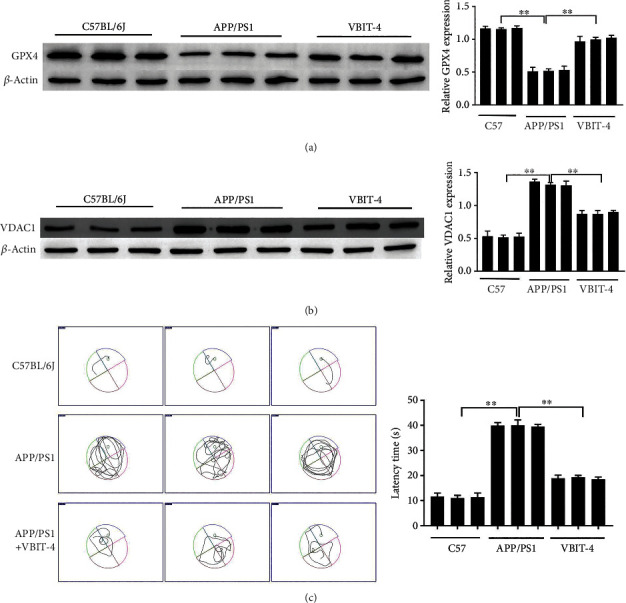
Inhibition of VDAC1 effects on cognitive function in APP/PS1 mice. The expressions of GPX4 and VDAC1 in tissues were measured via western blotting (a, b). Representative images of the escape track of rats in the Morris water maze test (c). Asterisks indicate statistical significance (^∗^*p* < 0.05, ^∗∗^*p* < 0.01).

VBIT-4 is a VDAC1 oligomerization inhibitor for the treatment of apoptosis-associated diseases such as neurodegenerative and cardiovascular diseases [36]. The results of the treatment of APP/PS1 mice with VBIT-4 showed that VDAC1 was significantly upregulated in APP/PS1 mice and decreased after VBIT-4 treatment. The expression of GPX4 declined in APP/PS1 mice, which was restored in VBIT-4 treatment (Figures 5(a) and 5(b)). In addition, to elucidate the effect of VDAC1 on learning and memory in APP/PS1 mice, the MWM test was performed. The MWM test showed that APP/PS1 mice exhibited a longer latency to find the platform; VBIT-4 treatment significantly reduced the latency to find the platform in APP/PS1 mice (Figure 5(c)).

### 3.6. Effects of Inhibition of VDAC1 and Ferroptosis on A*β*_1-42_-Induced AMPK/mTOR Pathway

**Figure 6:**
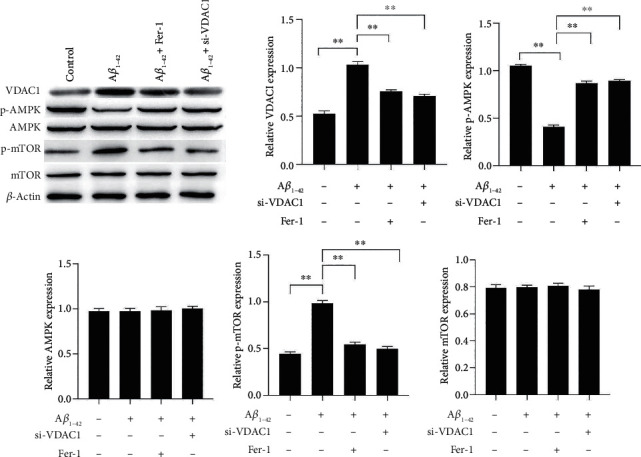
Effects of inhibition of VDAC1 and ferroptosis on the A*β*_1-42_-induced AMPK/mTOR pathway. The expressions of VDAC1, p-AMPK/AMPK, and p-mTOR/mTOR in PC12 cells were measured via western blotting. Asterisks indicate statistical significance (^∗^*p* < 0.05, ^∗∗^*p* < 0.01).

Activation of AMPK prevents neuronal apoptosis and increases neuronal viability [37]. We validated the expression of the AMPK pathway using western blot in A*β*_1-42_-induced cells, after si-VDAC1 and Fer-1 treatment. The results showed that the A*β*_1-42_ treatment of cells inhibited the phosphorylation of AMPK and upregulated the expression of p-mTOR. In addition, si-VDAC1 and Fer-1 treatment restored AMPK phosphorylation and downregulated the expression of p-mTOR (Figure 6). These results suggested that si-VDAC1 activates the AMPK pathway with the same effect as Fer-1 treatment.

### 3.7. Effects of Inhibition of VDAC1 and Ferroptosis on A*β*_1-42_-Induced Wnt/*β*-Catenin Pathway

**Figure 7:**
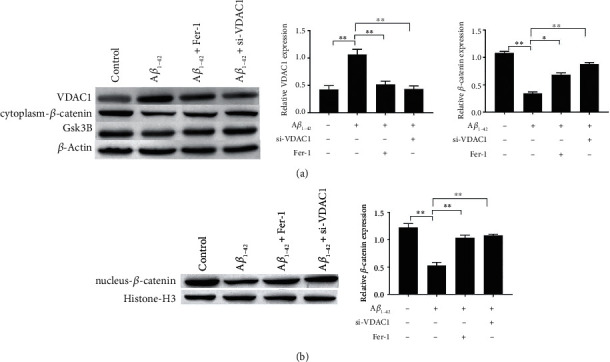
Effects of inhibition of VDAC1 and ferroptosis on A*β*_1-42_-induced Wnt/*β*-catenin pathway. The expressions of VDAC1 and *β*-catenin in PC12 cells were measured via western blotting. Asterisks indicate statistical significance (^∗^*p* < 0.05, ^∗∗^*p* < 0.01).

Next, we examined changes in the Wnt/*β*-catenin pathway. Western blotting results showed that the Wnt/*β*-catenin pathway-related protein *β*-catenin was downregulated in both the cytoplasm and nucleus in A*β*_1-42_-induced PC-12 cells. These results suggested that the A*β*_1-42_-inhibited Wnt/*β*-catenin pathway reduces the accumulation of *β*-catenin in the nucleus (Figures 7(a) and 7(b)). Furthermore, si-VDAC1 and Fer-1 treatment restored the expression of *β*-catenin (cytoplasmic and nuclear) (Figures 7(a) and 7(b)). These results suggested that si-VDAC1 activates the Wnt/*β*- catenin pathway with the same effect as Fer-1 treatment.

## 4. Discussion

Impaired cellular mitochondrial function and ferroptosis are widely reported in neurodegenerative diseases such as AD [38–42]. However, the underlying mechanisms and roles in regulating mitochondrial function and ferroptosis in AD are still unclear. In this study, we demonstrate that A*β*_1-42_ induced mitochondrial dysfunction and ferroptosis in PC12 cells (and SH-SY5Y cells); simultaneously, VDAC1 was highly expressed in the brains of AD mouse models and in A*β*_1-42_-induced PC12 cells. In addition,we noticed that Ferrostatin-1 and si-VDAC1 alleviated the downregulation of GPX4, FTH1 by A*β*_1-42_. Our result also showed that A*β*_1-42_ treatment of cells inhibited AMPK phosphorylation, which were reversed by Ferrostatin-1 and si-VDAC1. The present findings provide a molecular mechanism by which VDAC1 promotes mitochondrial dysfunction and ferroptosis by inhibiting AMPK/mTOR and Wnt/*β*-catenin to downregulate GPX4 in A*β*_1-42_-induced PC-12 cells.

A*β* accumulates in neurons in the AD patient brain. Simultaneously, elevated A*β* levels contribute to the mitochondrial abnormalities, producing ROS, which is the main pathogenesis of neurodegeneration [43]. Mitochondria are the major source of intracellular ROS; A*β* has been found in the mitochondrial membrane and interacts with mitochondrial proteins, affects the kinetics of mitochondrial fusion/fission [44], disrupts the electron transport chain and mitochondrial respiratory chain complex, increases the production and accumulation of ROS, and impairs mitochondrial function. Our results also confirmed it. Here, we found that A*β* enhanced ROS production and decreased the ΔΨm in PC12 cells and disrupted mitochondrial respiratory chain complexes I and IV. Excessive production of ROS in mitochondria can cause excessive mitochondrial damage and activate ferroptosis [45]. It has been reported that there are many ferroptosis-related features in AD [28]. Previous studies have found that iron exposure increases A*β* deposition and synaptic loss, exacerbating cognitive dysfunction in mice [46]. Although it has been demonstrated in some studies that A*β* accumulation may sequester iron and contribute to iron homeostasis in the brains of APP/PS1 mice [46, 47], this homeostasis will be disrupted as mice age and A*β* accumulates [46]. Reduced GPX4 activity is a hallmark of ferroptosis [48]. Our present results found that A*β*_1-42_ treatment decreased the expression levels of GPX4 and FTH1 in PC12 and SH-5HY cells. In addition, Ferrostatin-1 not only restored A*β*-induced GPX4 and FTH1 expression but also attenuated A*β*-induced mitochondrial dysfunction and decreased ΔΨm, ROS accumulation, and disruption of mitochondrial respiratory chain complexes I and IV. This result links mitochondrial dysfunction, ferroptosis, and AD progression.

VDAC1 is mainly expressed in the outer mitochondrial membrane, which participates in cellular metabolism and survival by regulating mitochondrial function. Due to the important role of mitochondrial function in AD, VDAC1 has been implicated in AD progression [49, 50]. After accumulation in the brain, A*β* enters mitochondria and interacts with VDAC1 to induce proapoptotic pores, resulting in the release of cytochrome c and activation of caspases [51, 52]. Consistent with a previous study [53], our results showed that the expression of VDAC1 is upregulated in A*β*_1-42_-treated PC12 and SH-SY5Y cells. In addition, VDAC1 has also been confirmed to be highly expressed in postmortem brain tissue from AD patients and also in a high level of A*β* transgenic mice [22]. However, none of the previous studies have inhibited VDAC1 in APP/PS1 mice to detect the changes in cognitive function of APP/PS1 mice. In this study, APP/PS1 mice were further treated with VDAC1 inhibitor VBIT-4, and the results of the water maze showed that cognitive function of APP/PS1 mice was improved after the action of VBIT-4. Interestingly, in the present study, si-VDAC1 not only rescued cell viability and decreased ROS but also restored the expression of GPX4 induced by A*β*_1-42_. This suggests that VDAC1 plays a critical role in promoting AD-related ferroptosis. Previous studies found that iron ions can enter mitochondria through VDAC1 on the mitochondrial outer membrane, causing iron accumulation and promoting cell damage [25, 54]. In neuronal cells, inhibition of VDAC1 was shown to prevent glutamate-induced ferroptosis and mitochondrial fragmentation [55]. These studies suggest that VDAC1 plays a key role in the progression of ferroptosis. In our study, Fer-1 restored A*β*-upregulated VDAC1. Simultaneously, in A*β*_1-42_-induced PC12 cells, si-VDAC1 treatment effects are the same as those of the Fer-1 treatment, both attenuating A*β*-induced mitochondrial dysfunction, ΔΨm reduction, ROS accumulation, and disruption of mitochondrial respiratory chain complexes I and IV. These results suggest an important role for inhibition of VDAC1 in alleviating A*β*_1-42_-induced mitochondrial dysfunction and ferroptosis. Our next step will be to explore the possible mechanism of action around VDAC1.

AMPK has been implicated with a variety of neurodegenerative diseases [56], with AMPK activation both promoting neuroprotection [57] and inducing neuronal death [58]. One possible explanation for this difference could be that AMPK plays different roles in neuronal damage over different time periods; that is, AMPK is activated during early neuronal injury and the p-AMPK/AMPK ratio is reduced as neuronal injury worsens [59, 60]. Of course, more research is needed to support this conclusion. At present, research on the protective effect of AMPK activation on neurons is still the focus. Treatment with metformin enhanced cerebral AMPK activation, meanwhile suppressing the activation of mTOR, attenuated spatial memory deficit, neuron loss in the hippocampus, and enhanced neurogenesis in APP/PS1 mice [61]. In addition, activation of AMPK prevents neuronal apoptosis and increases neuronal viability [37]. Meanwhile, activation of AMPK/mTOR induces autophagy and promotes neuronal survival during mitochondrial dysfunction [62]. In our current study, A*β*_1-42_ treatment of PC12 cells inhibited the phosphorylation of AMPK and upregulated the expression of p-mTOR. In addition, our results showed that si-VDAC1 and Fer-1 treatment restored AMPK phosphorylation and downregulated the expression of p-mTOR. Among them, the experimental results of VDAC1 and AMPK are consistent with the findings of Sarah et al. VDAC1 can act as a mediator of the AMPK/mTOR pathway to inhibit the phosphorylation of AMPK [63]. The results of this part of our study suggest that the cellular damage promoted by A*β*_1-42_ action through VDAC1 is associated with the inhibition of the AMPK/mTOR pathway. Previous studies have found that Wnt/*β*-catenin appears to be an important regulator of neurodegenerative diseases including AD. Because of the protective role of Wnt/*β*-catenin signaling in neurons, activation of Wnt/*β*-catenin signaling is considered to have potential value in the treatment of neurodegenerative diseases affecting synaptic integrity [64]. Previous studies have reported that *β*-catenin is downregulated in AD [65, 66], which is consistent with our findings in A*β*_1-42_-induced PC12 cells. In addition, it has been found that VDAC1 oligomerization inhibits the accumulation of nuclear *β*-catenin [67]. Meanwhile, our study found that treatment with si-VDAC1 and Fer-1 restored the expression of *β*-catenin in the cytoplasm and nucleus. Therefore, the ability to activate Wnt/*β*-catenin signaling may be another reason for si-VDAC1 and Fer-1 to alleviate AD.

This study has potential limitation: (1) This study only demonstrated the effect of VDAC1 on AD via mitochondrial dysfunction and ferroptosis at the cellular level but lacked validation at the animal level. (2) A*β* and tau protein deposition are currently considered as neuropathological markers of AD. While we only investigated the mechanism of VDAC1 under A*β* deposition, we did not explore the mechanism of VDAC1 changes in the context of tau aggregation.

**Figure 8:**
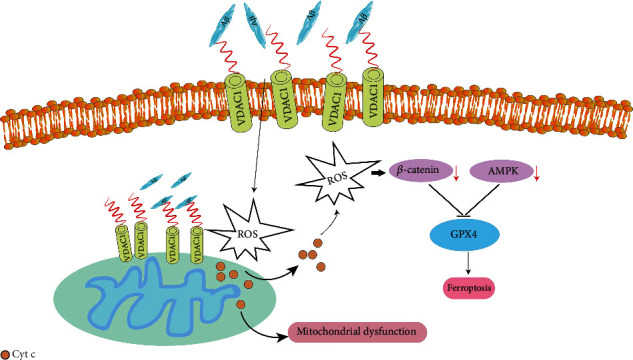
Schematic diagram of VDAC1 promotes mitochondrial dysfunction and ferroptosis by inhibiting AMPK/mTOR and Wnt/*β*-catenin.

In summary, our findings provide evidence to show that VDAC1 participates in A*β*_1-42_-induced neuronal mitochondrial dysfunction and ferroptosis and highlights the developmental by which AMPK/mTOR and Wnt/*β*-catenin are involved in this process (Figure 8). In conclusion, targeting VDAC1 to inhibit mitochondrial dysfunction and ferroptosis could be a promising therapeutic strategy for AD in the future.

To the best of our knowledge, this work provides the first description of VDAC1 in A*β*_1-42_-induced neuronal mitochondrial dysfunction and ferroptosis, which is associated with inhibition of AMPK/mTOR and Wnt/*β*-catenin pathways (Figure 8). Our results elucidate the importance of inhibiting VDAC1 in the development of AD to a certain extent and provide some support for later drug research targeting VDAC1 in the treatment of AD.

## Data Availability

The datasets used and/or analyzed during the current study are available from the corresponding author upon reasonable request.

## Supplementary Materials

Supplementary Materials A*β*_1-42_ effects on cell viability and cytotoxicity, and VDAC1, GPX4, and FTH1 expressions in SH-SY5Y cells. MTT and LDH assay results showed that Fer-1 treatment reduced cell death and LDH release (Figures S1A-S1B). Western blot analysis indicated that the A*β*_1-42_ treatment decreased the expression of GPX4 and FTH1 (Figure S1C). In addition, A*β*_1-42_ upregulated the expression of VDAC1 in SH-SY5Y cells. Fer-1 could inhibit the expression of VDAC1 increased by A*β*_1-42_ (Figure S1D). Figure S1: A*β*_1-42_ effects on cell viability and cytotoxicity and VDAC1, GPX4, and FTH1 expressions in SH-SY5Y cells. SH-SY5Y cell viability was measured via the MTT assay (a). The LDH release was measured via the LDH assay kit (b). The expressions of GPX4, FTH1, and VDAC1 in SH-SY5Y cells were measured via western blotting (c, d). Asterisks indicate statistical significance (^∗^*p* < 0.05, ^∗∗^*p* < 0.01).
