# Supplementary material for: Inhibition of VDAC1 Rescues Aβ1-42-Induced Mitochondrial Dysfunction and Ferroptosis via Activation of AMPK and Wnt/β-Catenin Pathways
Source: Mediators Inflamm. 2023 Feb 10;2023:6739691. doi: 10.1155/2023/6739691 (PMC9937775; doi:10.1155/2023/6739691)
Supplement: Supplementary Materials — Aβ1-42 effects on cell viability and cytotoxicity, and VDAC1, GPX4, and FTH1 expressions in SH-SY5Y cells. MTT and LDH assay results showed that Fer-1 treatment reduced cell death and LDH release (Figures S1A-S1B). Western blot analysis indicated that the Aβ1-42 treatment decreased the expression of GPX4 and FTH1 (Figure S1C). In addition, Aβ1-42 upregulated the expression of VDAC1 in SH-SY5Y cells. Fer-1 could inhibit the expression of VDAC1 increased by Aβ1-42 (Figure S1D). Figure S1: Aβ1-42 effects on cell viability and cytotoxicity and VDAC1, GPX4, and FTH1 expressions in SH-SY5Y cells. SH-SY5Y cell viability was measured via the MTT assay (a). The LDH release was measured via the LDH assay kit (b). The expressions of GPX4, FTH1, and VDAC1 in SH-SY5Y cells were measured via western blotting (c, d). Asterisks indicate statistical significance (∗p < 0.05, ∗∗p < 0.01). [file 6739691.f1.zip › WB.docx]

Fig1C-GPX4:


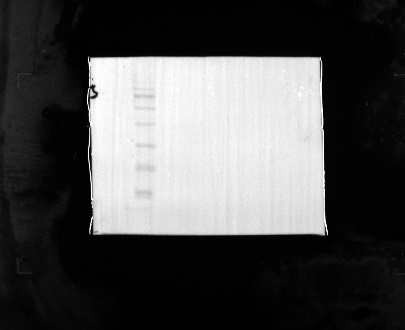



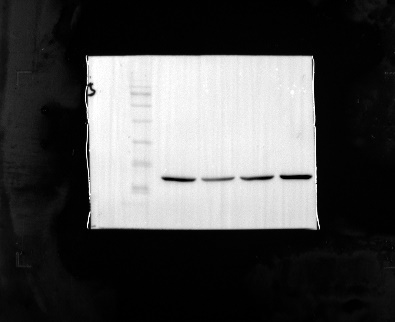


Fig1C-FTH1:


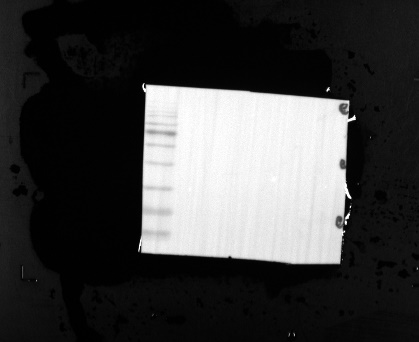

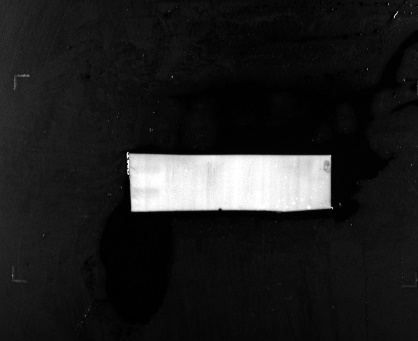




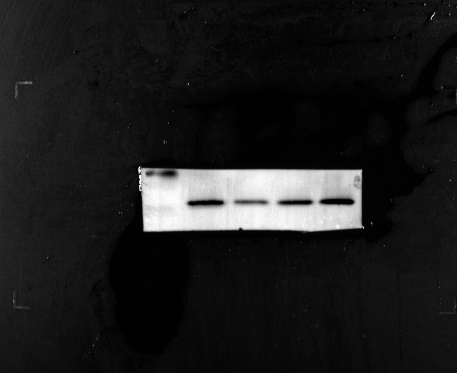


Fig1C-β-actin:


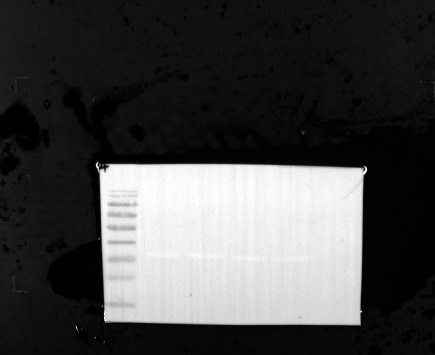



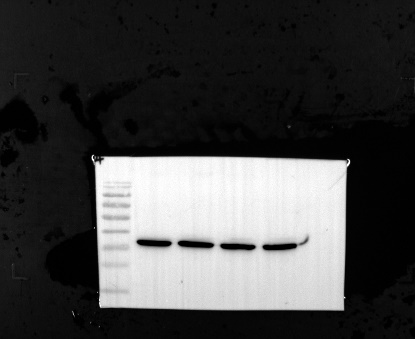


Fig1D-VDAC1:


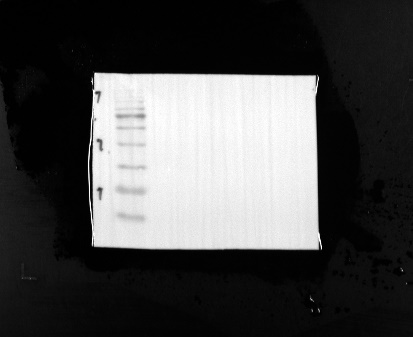

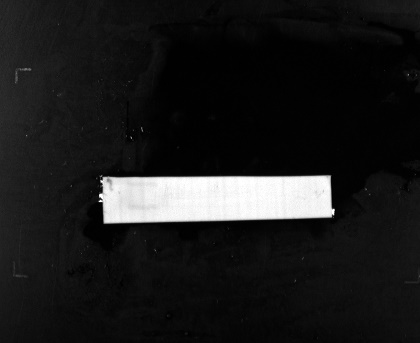



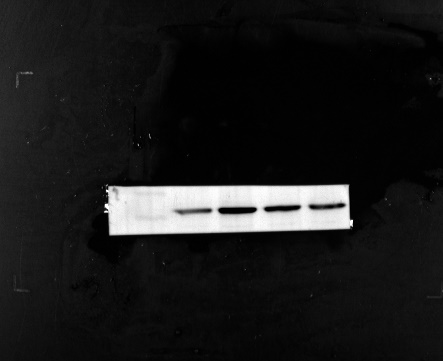


Fig1D- β-actin:


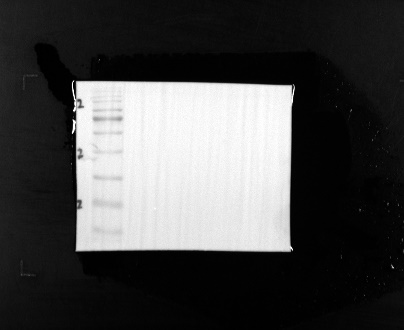

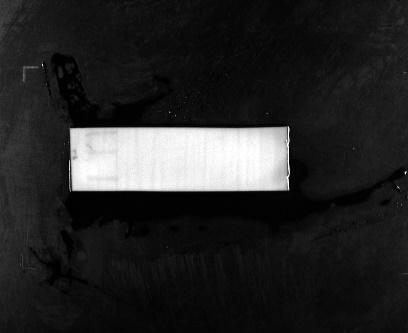



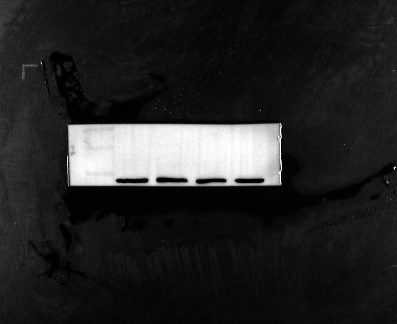


FigS1C-GPX4:









FigS1C-FTH1:


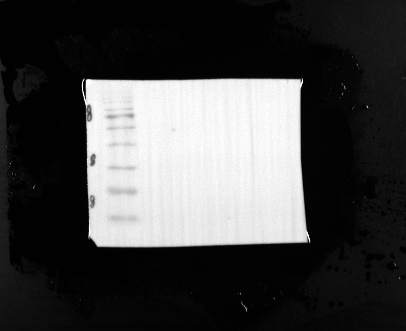

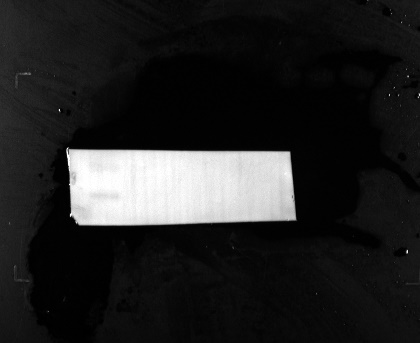



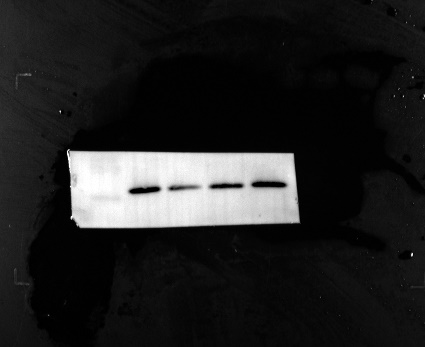


FigS1C- β-actin:









FigS1D- VDAC1:


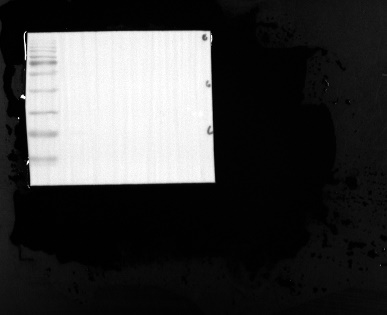

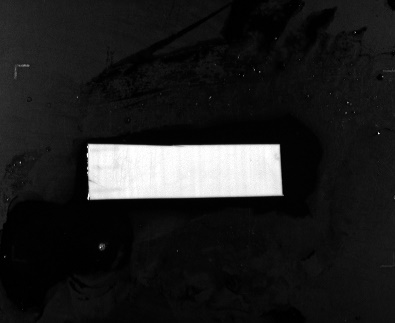



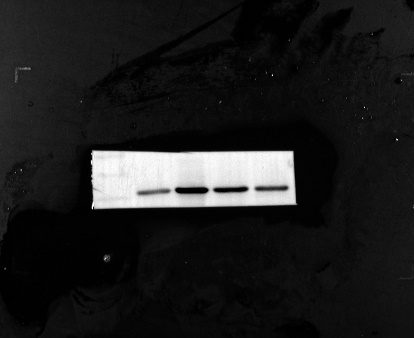


FigS1D- β-actin:


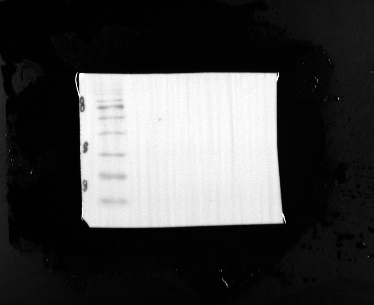

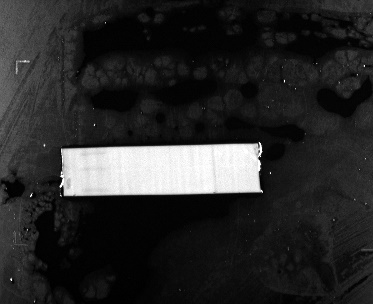



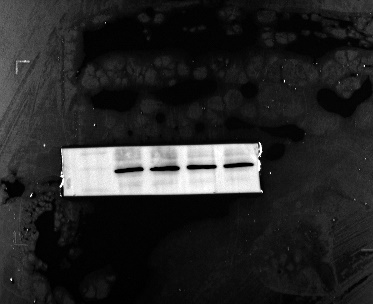


Fig 3A-VDAC1：


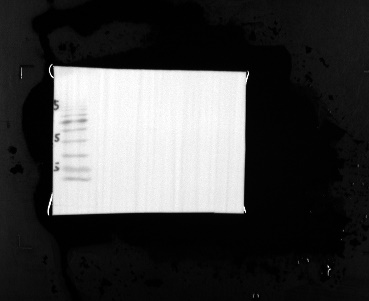

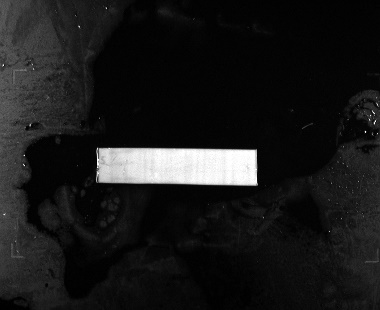




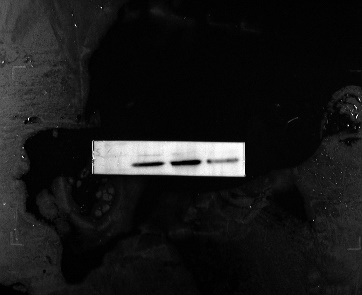


Fig 3A- β-actin：


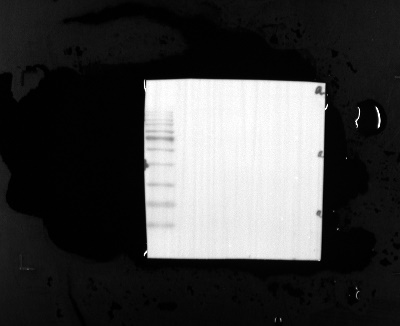

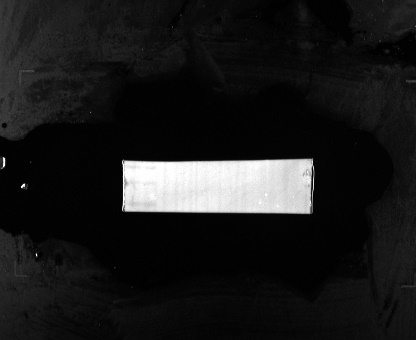




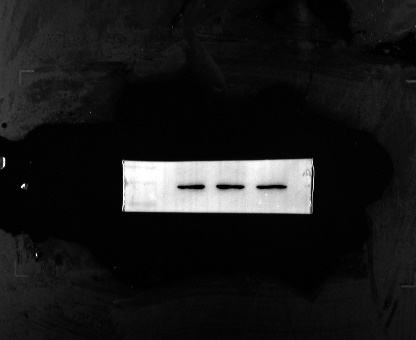


Fig 3D- GPX4：


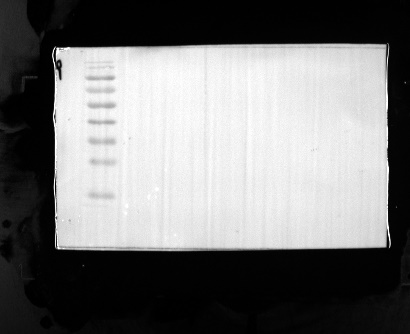



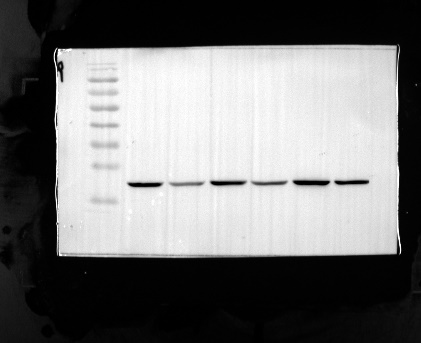


Fig 3D- FTH1：


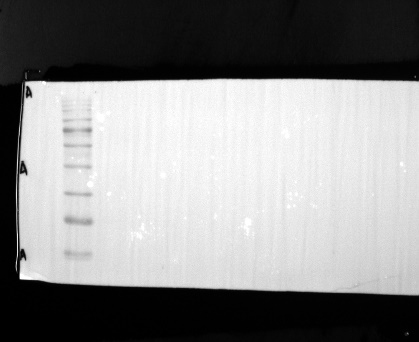









Fig 3D- β-actin：


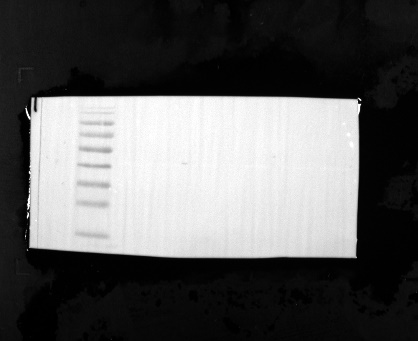



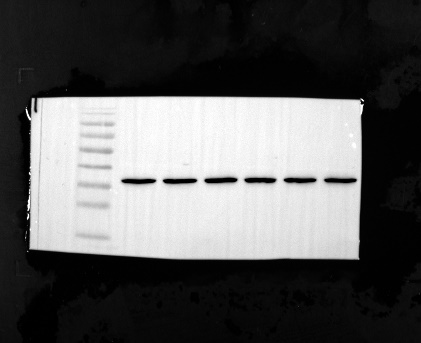


Fig 5A- GPX4：


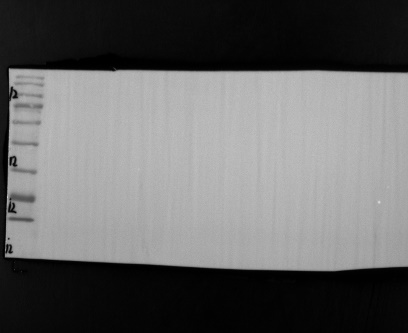









Fig 5A- β-actin：


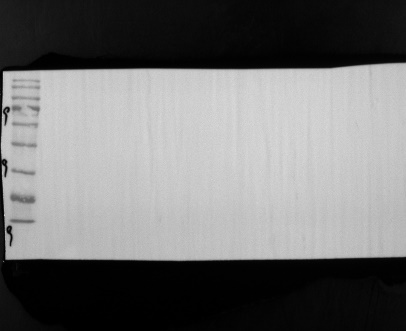









Fig 5B- VDAC1：


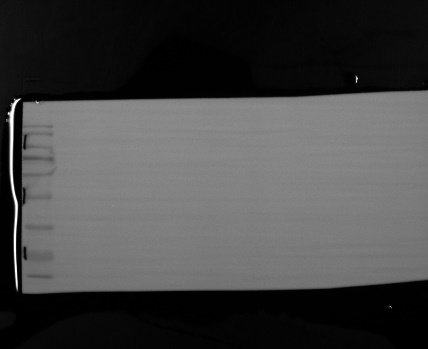









Fig 5B- β-actin：


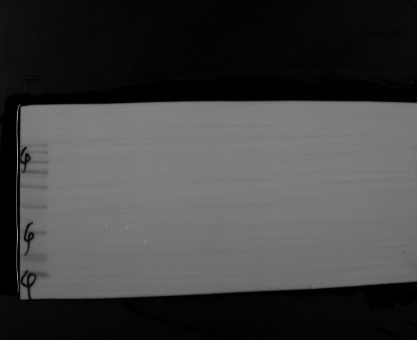









Fig 6-VDAC1:


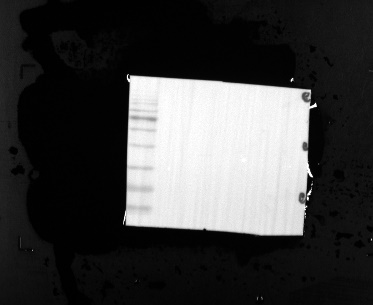

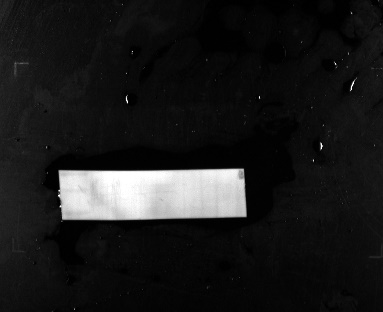




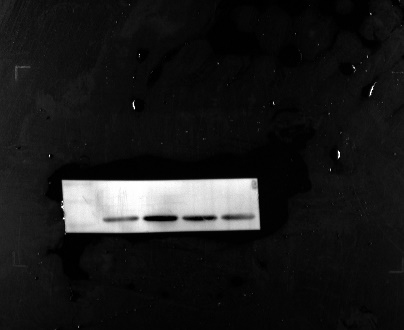


Fig 6-pAMPK:


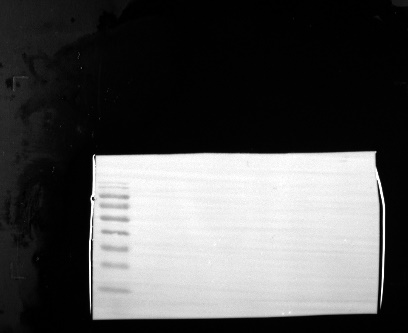



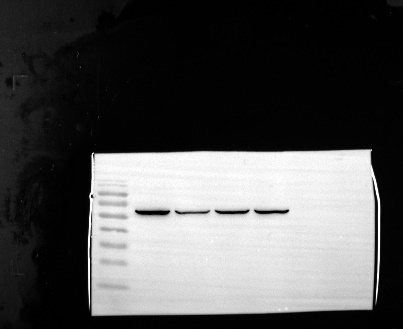


Fig 6-AMPK:


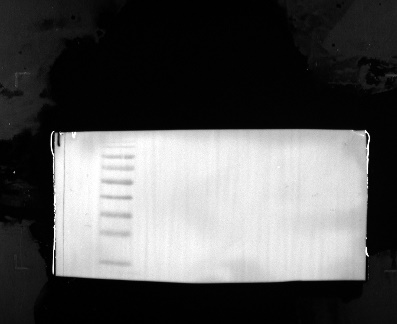



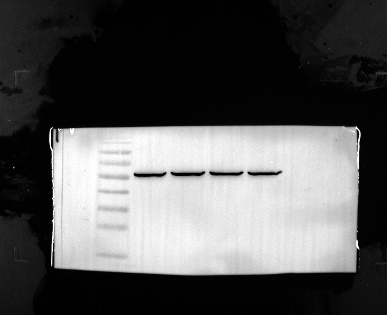


Fig 6-p-mTOR:


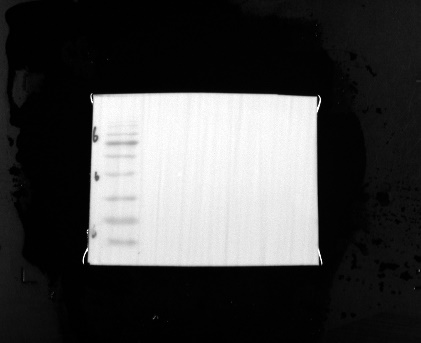

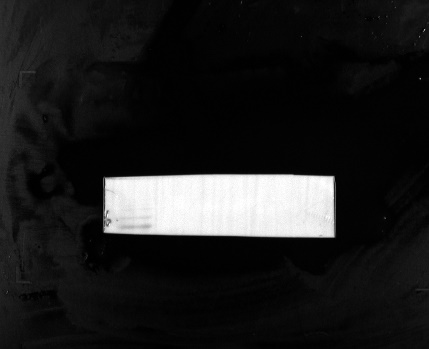



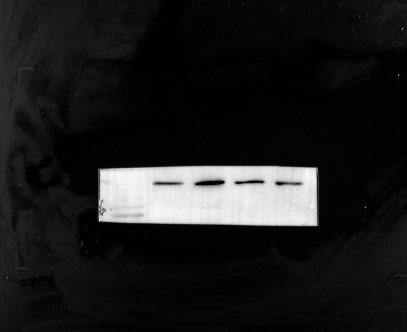


Fig 6-mTOR:


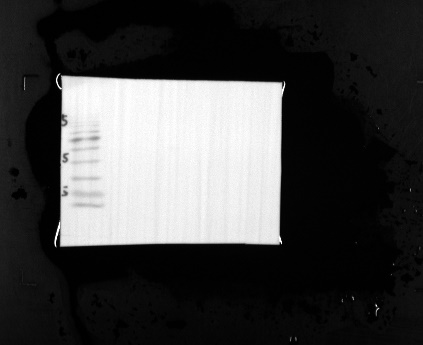

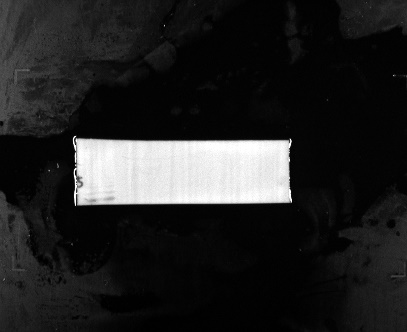



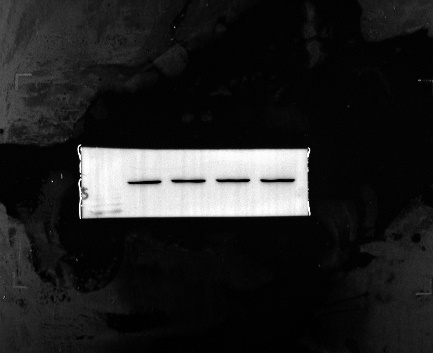


Fig 6- β-actin:


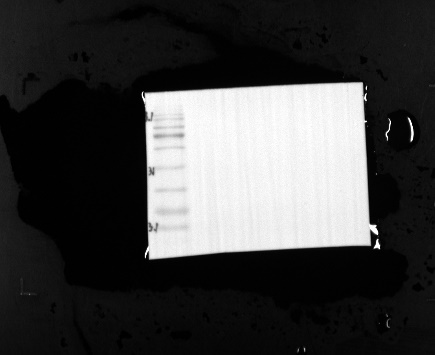

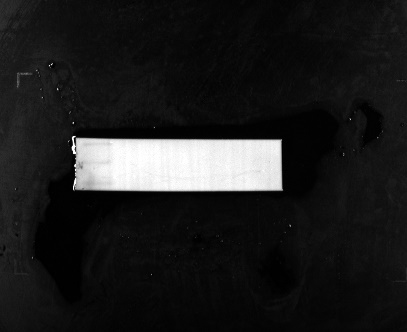




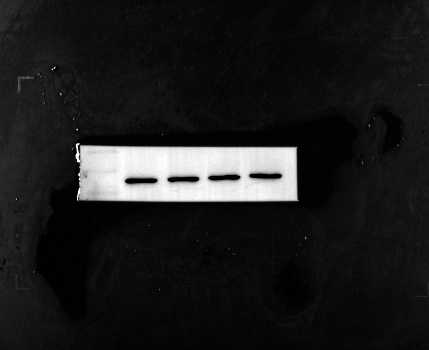


Fig 7A-VDAC1


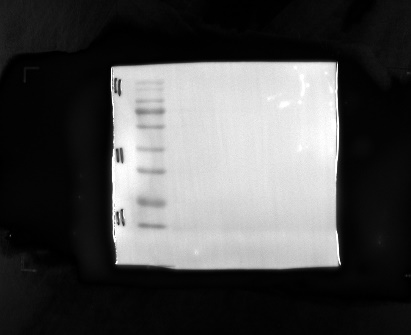








Fig 7A- β-catenin


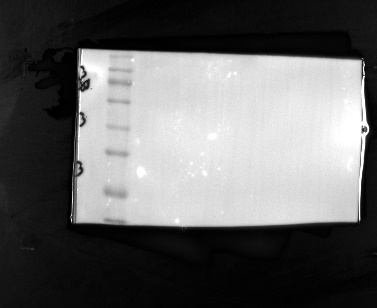








Fig 7A- Gsk3B

Fig 7A- β-actin

Fig 7B- n-β-catenin

Fig 7B- Histone-H3
